# Supplementary material for: Heme controls the structural rearrangement of its sensor protein mediating the hemolytic bacterial survival
Source: Commun Biol. 2021 Apr 13;4:467. doi: 10.1038/s42003-021-01987-5 (PMC8044140; doi:10.1038/s42003-021-01987-5)
Supplement: Supplementary file 1 — Supplementary Information [file 42003_2021_1987_MOESM1_ESM.pdf]

Supplementary Information

(Supplementary Figures 1-12 and Tables 1)

Heme Controls the Structural Rearrangement of Its Sensor Protein Mediating the Hemolytic Bacterial Survival

Megumi Nishinaga[1], Hiroshi Sugimoto[1,2], Yudai Nishitani[1], Seina Nagai[1], Satoru Nagatoishi[3], Norifumi Muraki[4], Takehiko Tosha[1,2], Kouhei Tsumoto[3,5,6], Shigetoshi Aono[4], Yoshitsugu Shiro[1,*] Hitomi Sawai[1,2,*]

[1]Graduate School of Life Science, University of Hyogo, 3-2-1 Kouto, Kamigori, Ako, Hyogo 679-1297, Japan

[2]RIKEN SPring-8 Center, 1-1-1 Kouto, Sayo, Hyogo 679-5148, Japan

[3]The Institute of Medical Science, The University of Tokyo, Minato-ku, Tokyo 108-8639, Japan

[4]Institute of Molecular Science, National Institute of Natural Sciences, Okazaki, Aichi 444-8787, Japan

[5]Department of Bioengineering, School of Engineering, The University of Tokyo, Minato-ku, Tokyo 108-8639, Japan

[6]Department of Chemistry and Biotechnology, School of Engineering, The University of Tokyo, Minato-ku, Tokyo 108-8639, Japan

[*]To whom correspondence should be addressed: 3-2-1 Kouto, Kamigori, Ako, Hyogo 679-1297, Japan (Y.S. and H.Sawai); E-mail: yshiro@sci.u-hyogo.ac.jp, Tel. +81-791-58-0325 (Y.S.); E-mail: sawai@sci.u-hyogo.ac.jp, Tel. +81-791-58-0412 (H.Sawai)

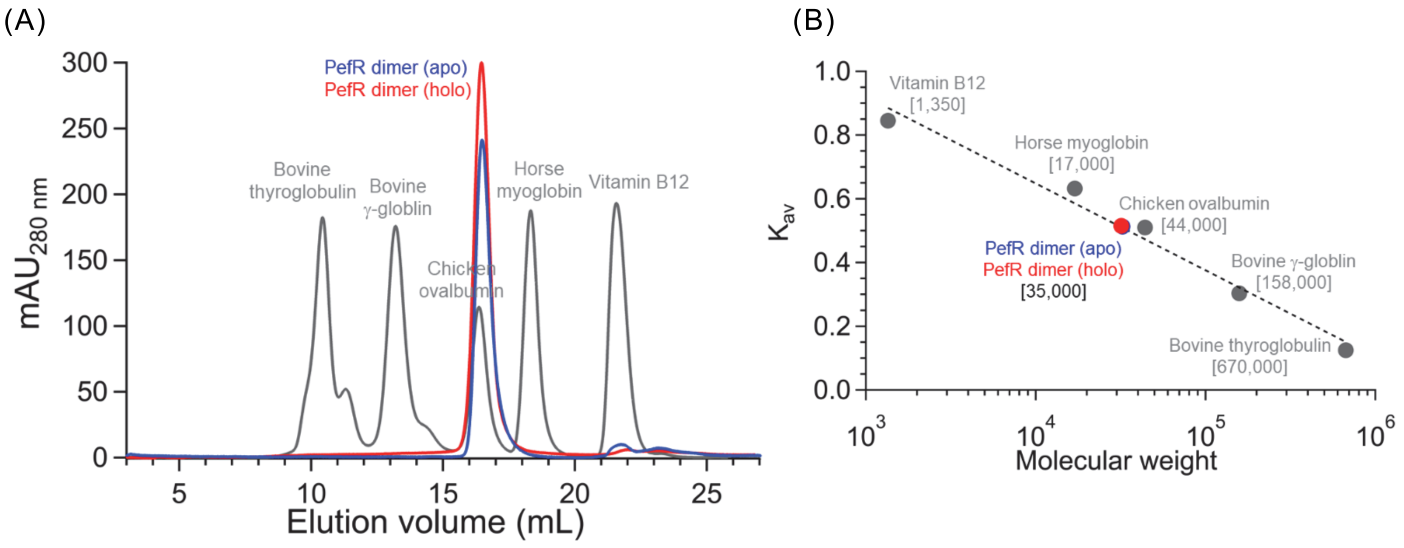

Supplementary Figure 1: Gel filtration profile (A) of purified PefR protein in apo and holo forms. (B) For molecular weight estimation, a calibration curve of K_{av} versus log molecular weight (inset, dotted line) was prepared using a commercially available gel filtration standard. Calculated K_{av} values were used $K_{av} = (V_e - V_0)/(V_c - V_0)$, where V_0 = column void volume, V_e = elution volume at the peak, and V_c = geometric column volume. The calibration curve provided the equation $Y = -0.272 \log X + 1.74$ to estimate the molecular weight (X) of PefR using Y (K_{av} value).

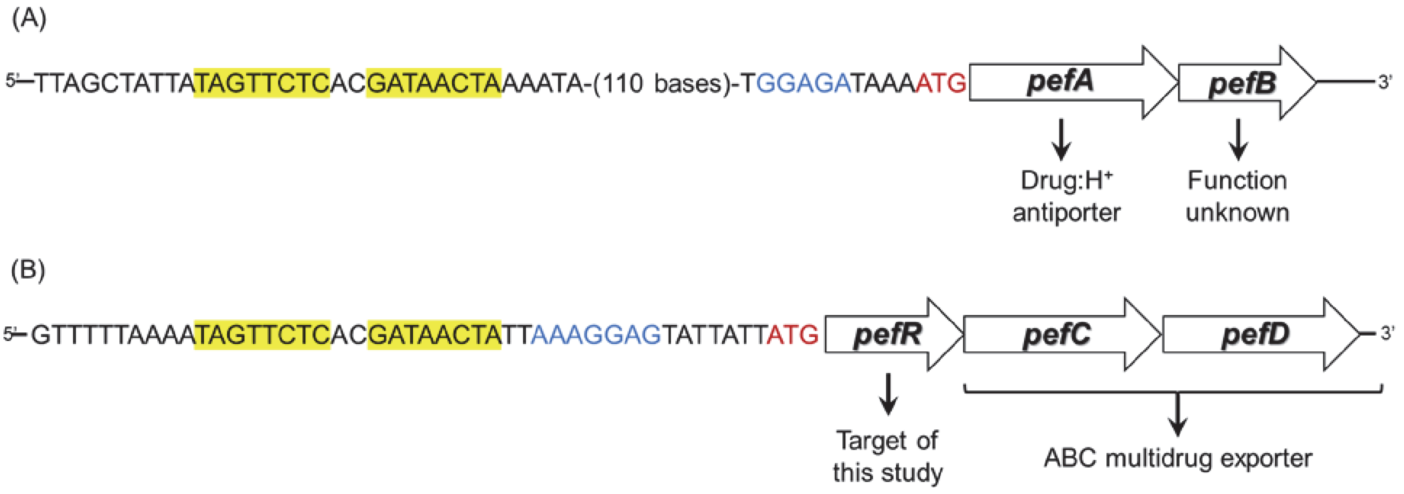

Supplementary Figure 2: Genetic context of two operons for the transcriptional regulation of PefR. (A) The *pefA* (*gbs1753*) and *pefB* (*gbs1752*) genes, and (B) the *pefR* (*gbs1402*) *pefC* (*gbs1401*) *pefD* (*gbs1400*) genes are represented by bold arrows (gene symbols are noted in brackets). The predicted products are labeled below the arrows. The inverted repeat motifs are highlighted in yellow. Ribosome binding sites and start codons are colored blue and red, respectively.

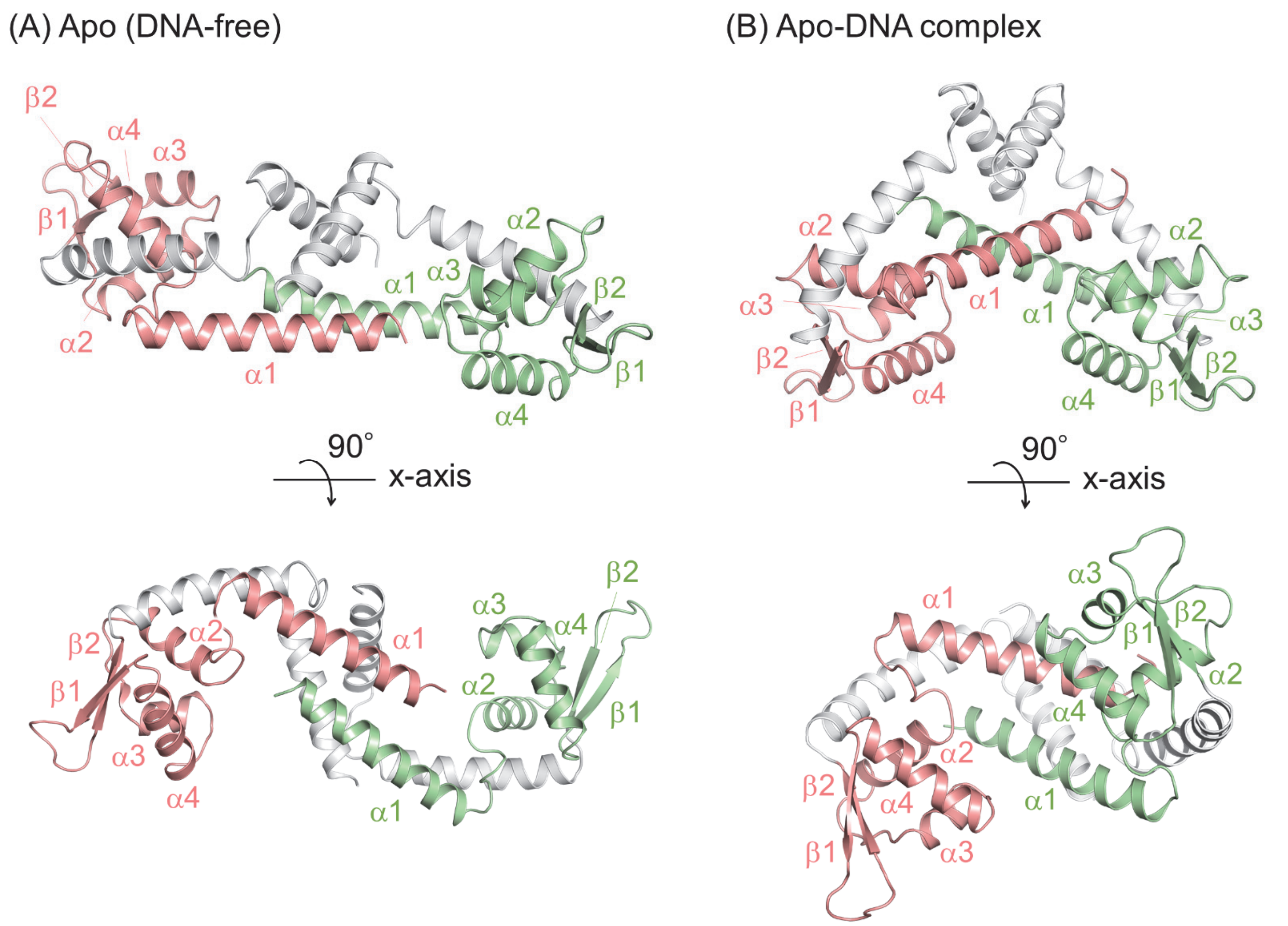

Supplementary Figure 3: Comparison of protein structures of apo-PefR with N-termial His-tag (A) and apo-PefR (non-tagged) in the vicinity of the bound DNA (B). α1 helices and DNA binding domain (α2-4 helices and β1-2 strands) are colored pink and green in each subunit of the dimer. Upper panels are views from the side, and bottom panels are views from the bottom, which are rotated 90° on the X-axis from their side views.

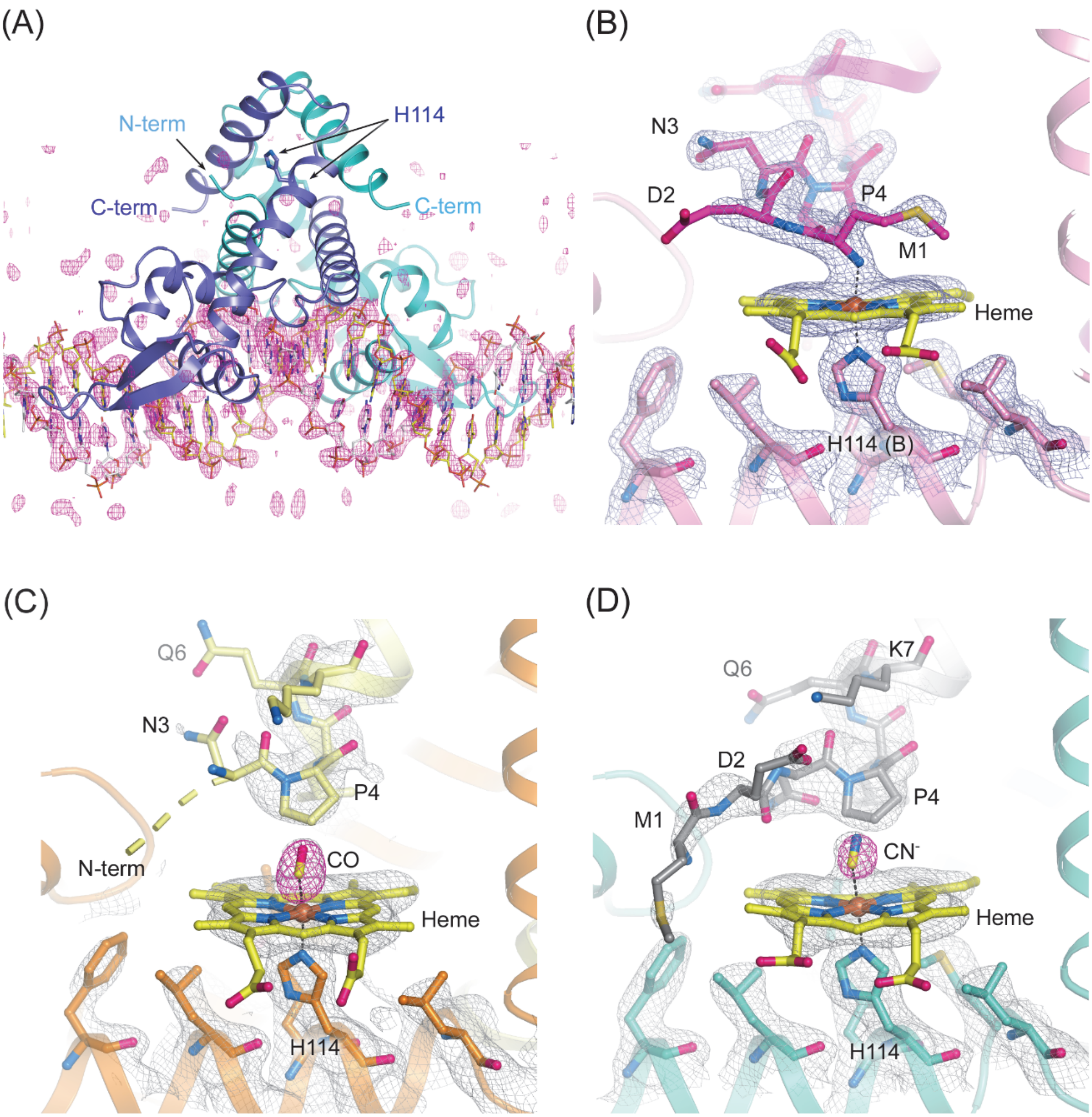

Supplementary Figure 4: Electron density map of PefR. (A) The overall crystal structure of the PefR-DNA complex demonstrates that the DNA duplex has well defined electron density, as shown by the omit map at 2.5 σ. (B) The electron density ($2F_o - F_c$ map) of the holo form of PefR in the region around the heme is contoured at 1.4 σ. (C) The $2F_o - F_c$ map of the heme and ligand binding site is shown at 1.1 σ as grey mesh and the omit map at 3.5 σ of the carbon monoxide (CO) is shown as magenta mesh. The atomic models for N-terminal residues 1–3 could not be built because of disordered electron density. (D) The omit map at 2.5 σ of the cyanide (CN^-) is shown as magenta mesh.

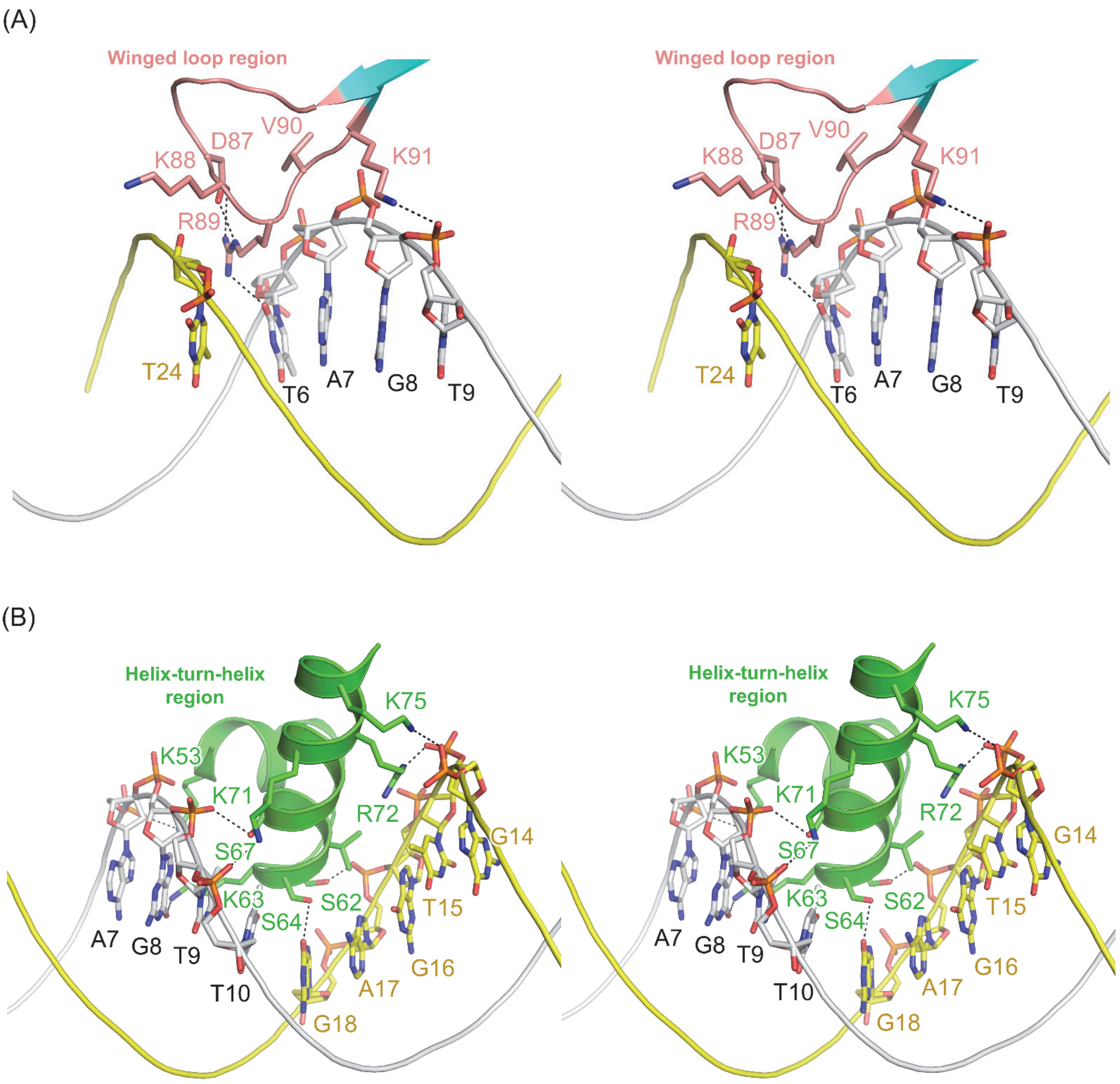

Supplementary Figure 5: Stereo-views (wall-eyes) of the interactions in the wHTH region of apo-PefR complexed with the target DNA. (A) Interaction between the winged loop region (pink) and the target DNA. (B) Interactions between the helix-turn-helix region (green) and the target DNA.

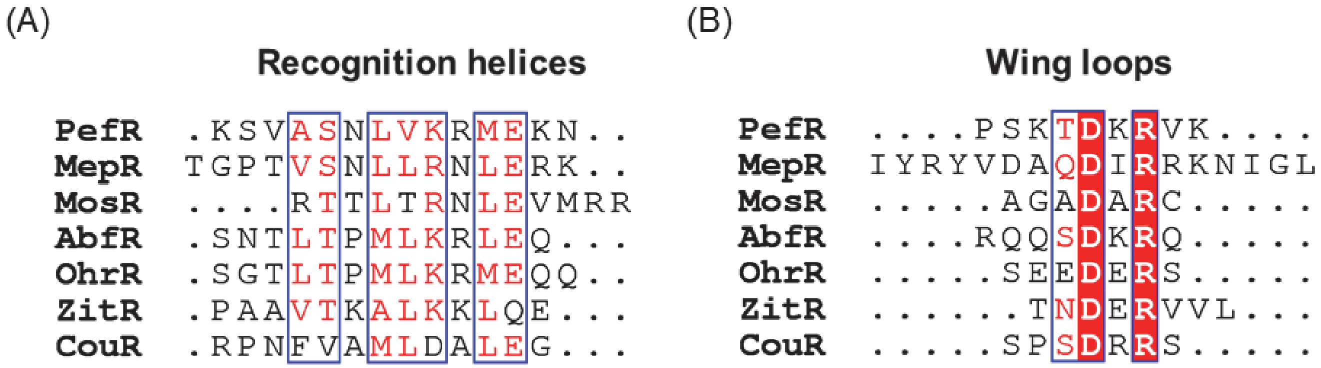

Supplementary Figure 6: Sequence alignment of (A) the recognition helix and (B) the wing motif region in the wHTH motif of PefR and other MarR superfamily proteins whose crystal structures complexed with DNA have been solved. Secondary structures of these sequences were assigned according to information in the Protein Data Bank. The alignments were created using ClustalW[1] and drawn with ESPript[2].

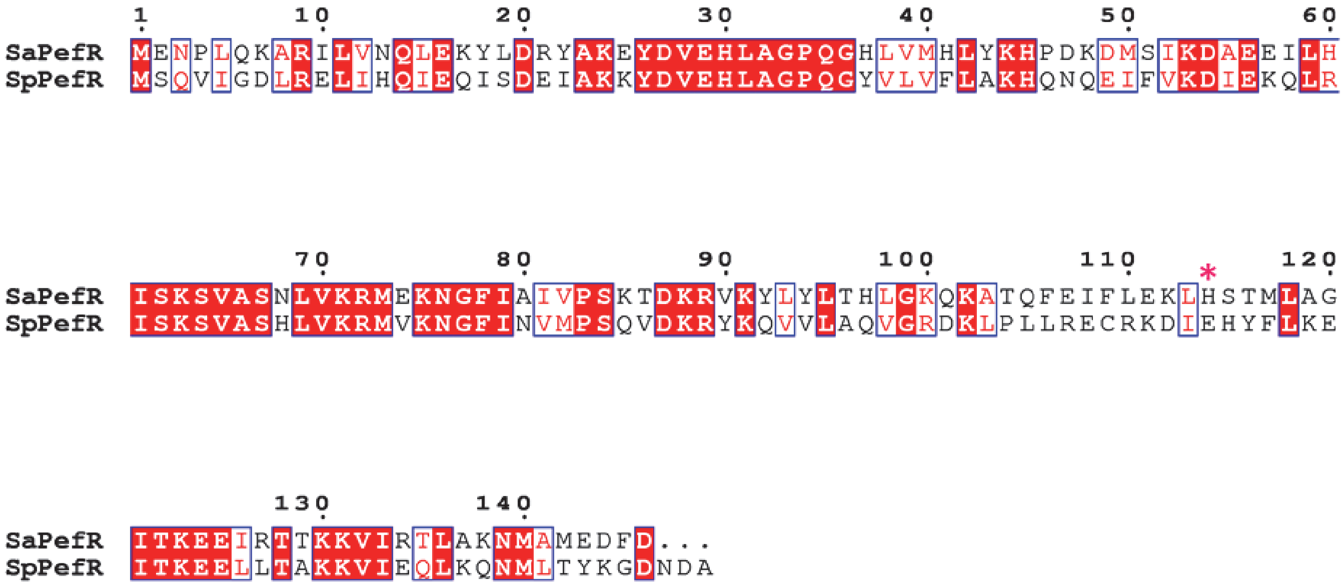

Supplementary Figure 7: Sequence alignment of PefR from *S. agalactiae* (SaPefR) and *S. pyogenes* (SpPefR). These amino acid sequences were assigned as gbs1402 (SaPefR) and WP_002986120 in the NCBI database. The heme axial His114 in SaPefR is marked with a pink asterisk. The alignments were created using ClustalW[1] and drawn with ESPript[2]. Our crystallographic and spectroscopic data clearly showed that *S. agalactiae* PefR combines with one heme molecule per monomer to form a 1:1 stoichiometric complex. In contrast, Sachla *et al.* reported that PefR from *Streptococcus pyogenes* (a Group A *Streptococcus*) binds two heme molecules to one protein (heme:PefR = 2:1). Indeed, the Soret peak positions in the absorption spectra of the holo forms (407 nm for *S. agalactiae* PefR and 435 nm for *S. pyogenes* PefR) differed from one another[1], although the tertiary structure of *S. pyogenes* PefR has yet to be reported. One possible explanation for this difference might be differences in sample preparation: *S. pyogenes* PefR was prepared as a fused form with the N-terminal His tag, and the His-tagged protein was used for the heme titration experiment. In this study, we found that the N-terminal region was important for heme binding and thus the N-terminal His tag might affect heme binding to *S. pyogenes* PefR. Alternatively, it is interesting that although the two PefRs share amino acid sequences with 43% identity and 74% similarity, His114 in the α5 helix, a heme axial residue in *S. agalactiae* PefR, is replaced with Glu114 in *S. pyogenes* PefR, and other residues in this helix are not well conserved in PefR.

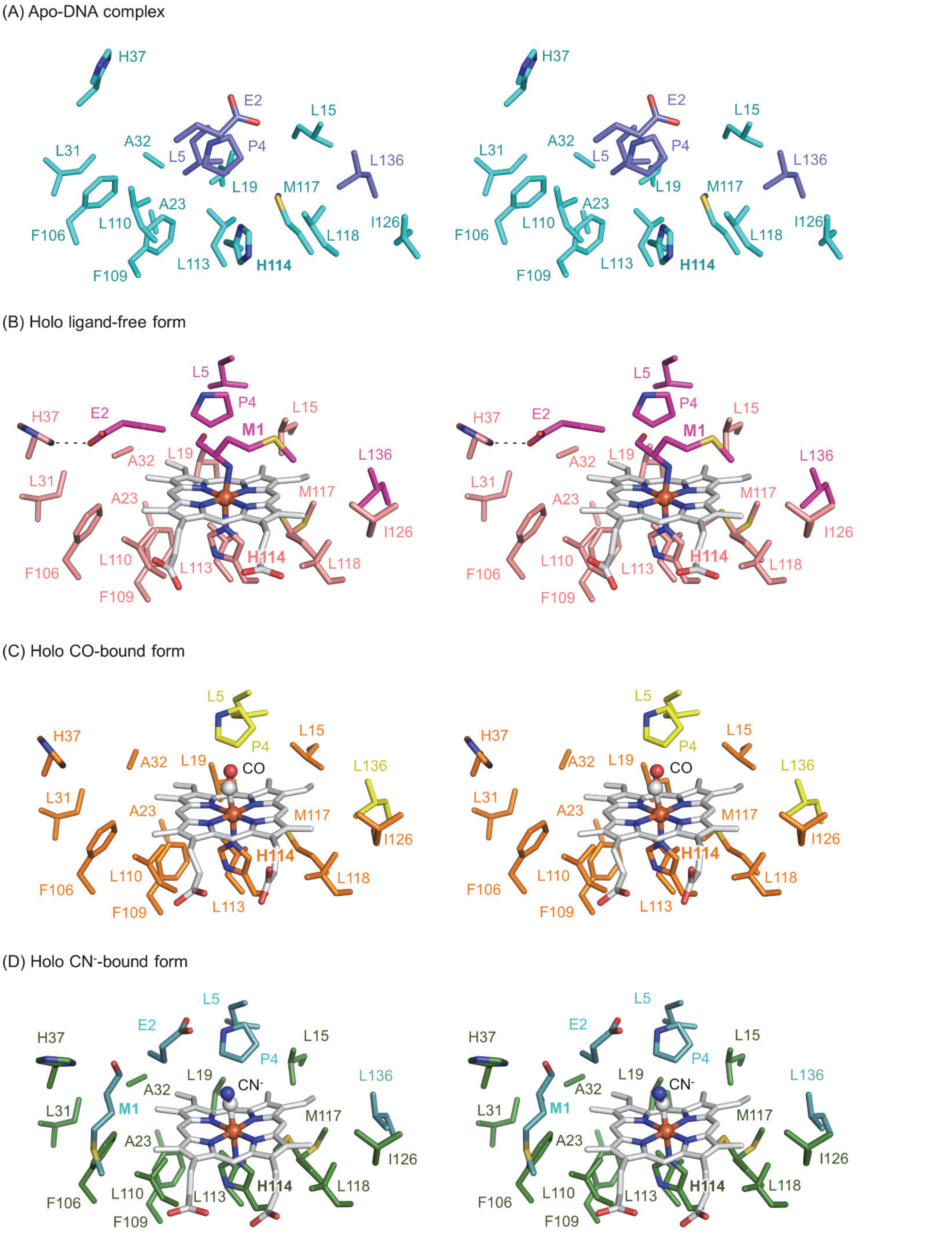

Supplementary Figure 8: Stereo-views (wall-eyes) of the structures in the vicinity of the heme of the apo (A), holo (B), CO-bound (C), CN-bound (D) forms.

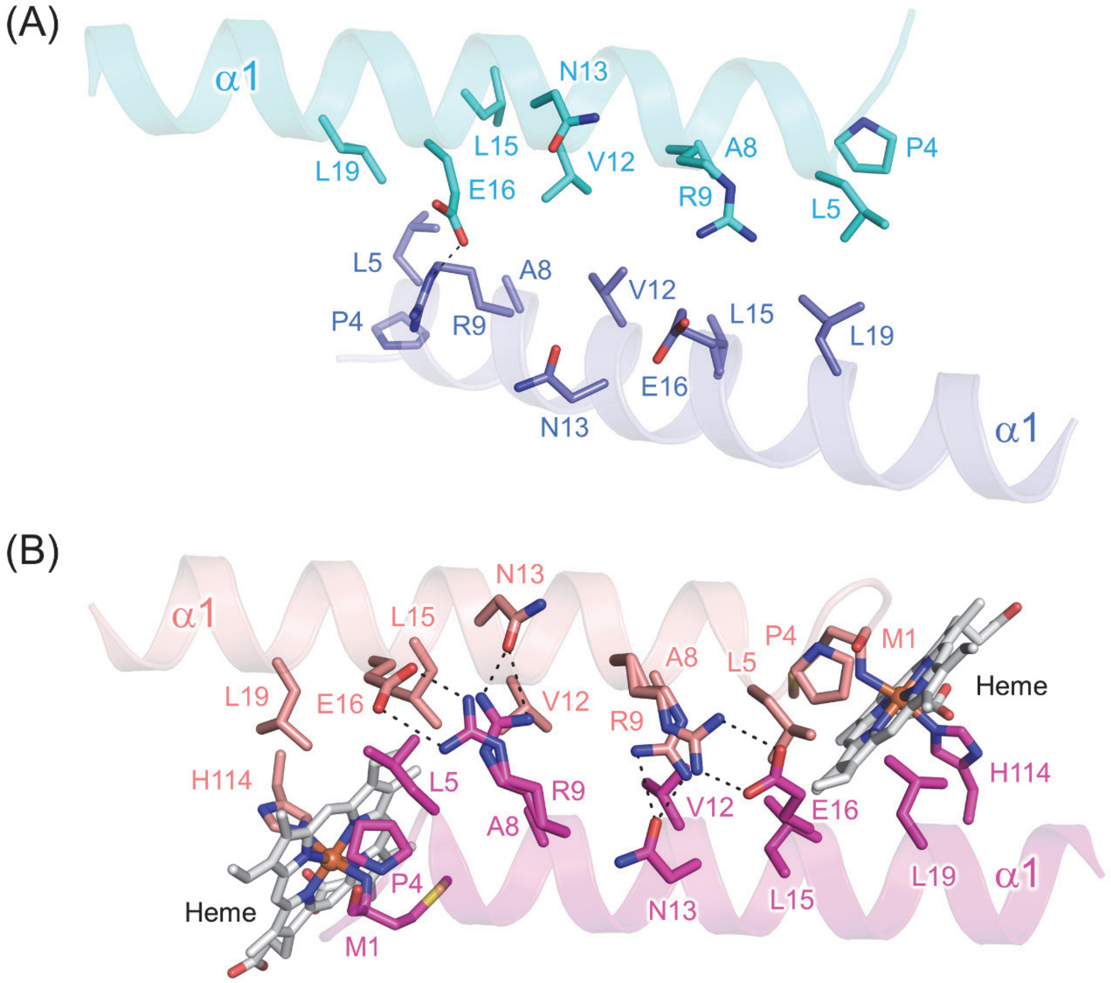

Supplementary Figure 9: Dimer interface between the α1 helices of the apo-PefR-DNA complex (A) and the holo form (B) in the homodimer. The colorings are the same as in Figures 2 and 5. Heme molecules and amino acid residues involved in van der Waals and polar interactions and heme coordination are drawn as sticks. The polar interactions of amino acid residues between each α1 helix are shown as dotted lines.

(A)

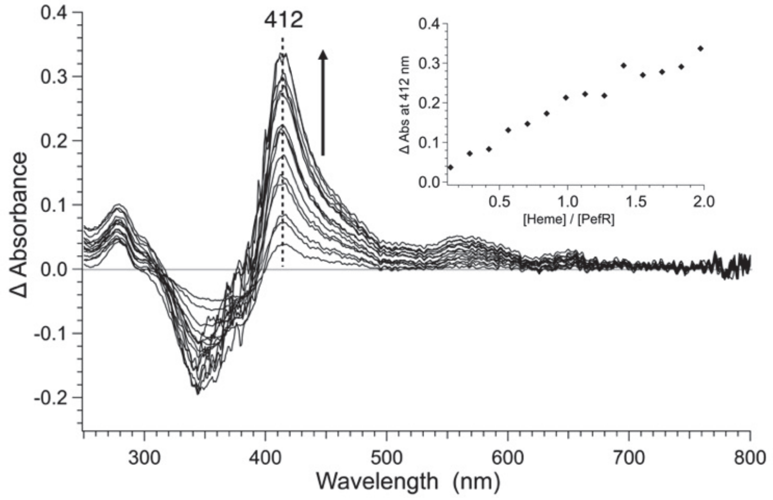

(B)

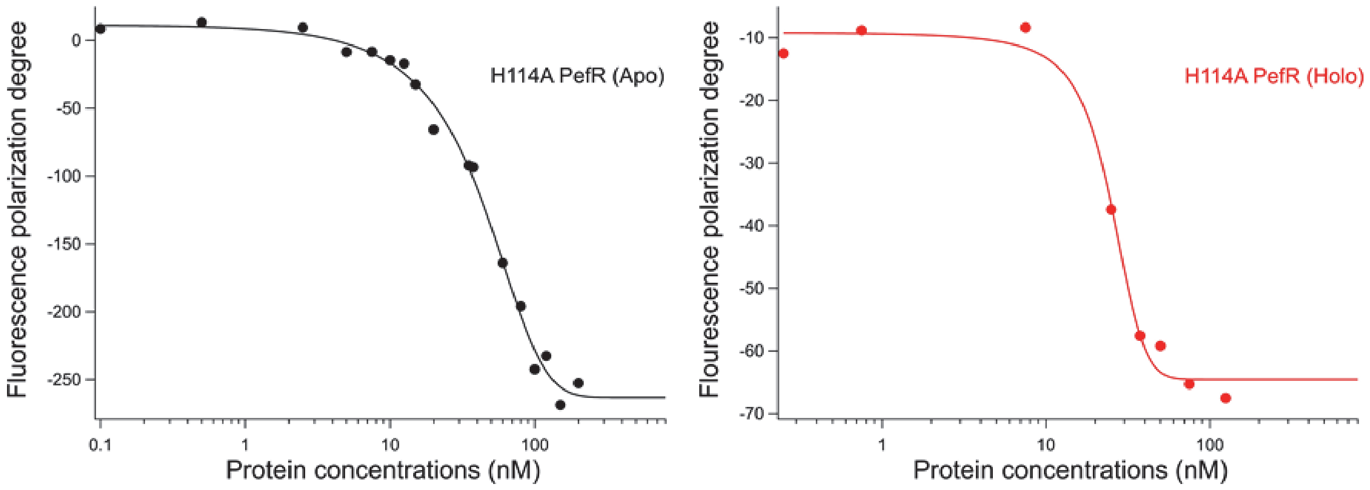

Supplementary Figure 10: Properties of H114A PefR. (A) Spectral change upon ferric heme addition. These spectra are similar to the spectrum of hemin, indicating that the H114A mutant does not bind heme. (B) Fluorescence polarization assays with apo (left panel) and holo (hemin added) (right panel) PefR proteins. The curve fittings indicate a K_d of *ca.* 25 nM, assuming a 1:1 binding ratio of apo-PefR dimer and one double-stranded DNA. This result indicates that H114A PefR can bind to the target DNA.

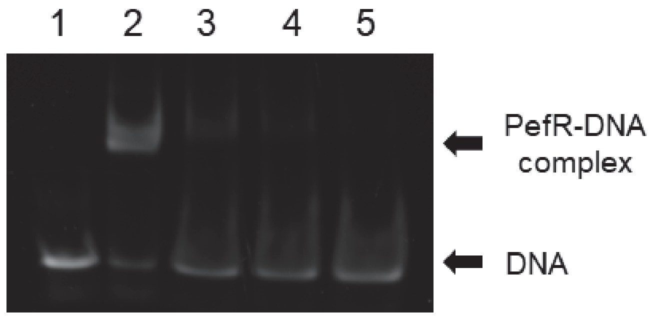

Supplementary Figure 11: EMSA assays for DNA binding with/without hemin and KCN addition. Lane 1: target DNA (5 μM), lane 2: 1 + 1 mol eq of apo-PefR in the dimer (5 μM), lane 3: 2 + 2.5 μM hemin + 1 mM KCN, lane 4: 2 + 5 μM hemin + 1 mM KCN, lane 5: 2 + 10 μM hemin + 1 mM KCN. Electrophoresis was performed with buffer containing 50 mM KCN.

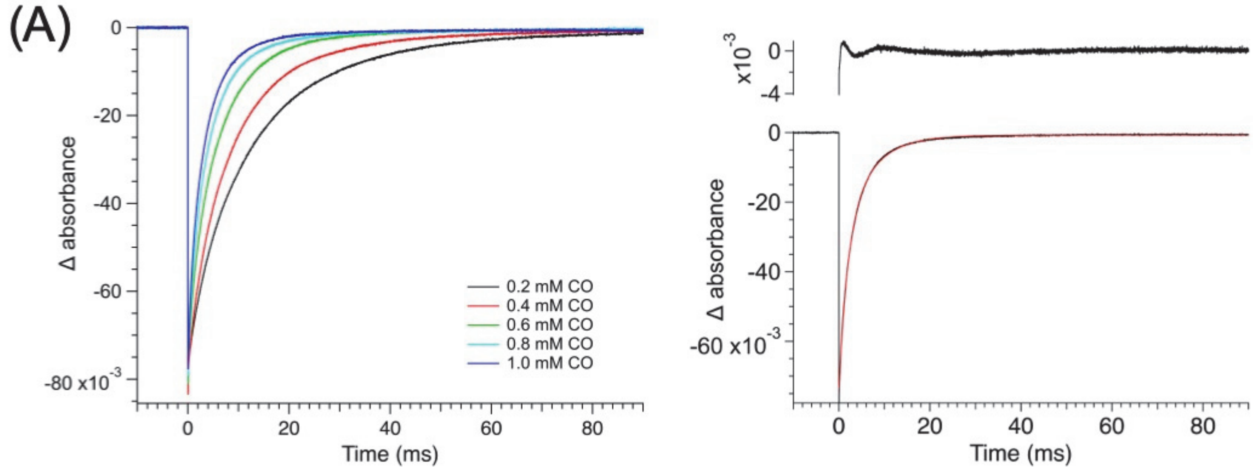

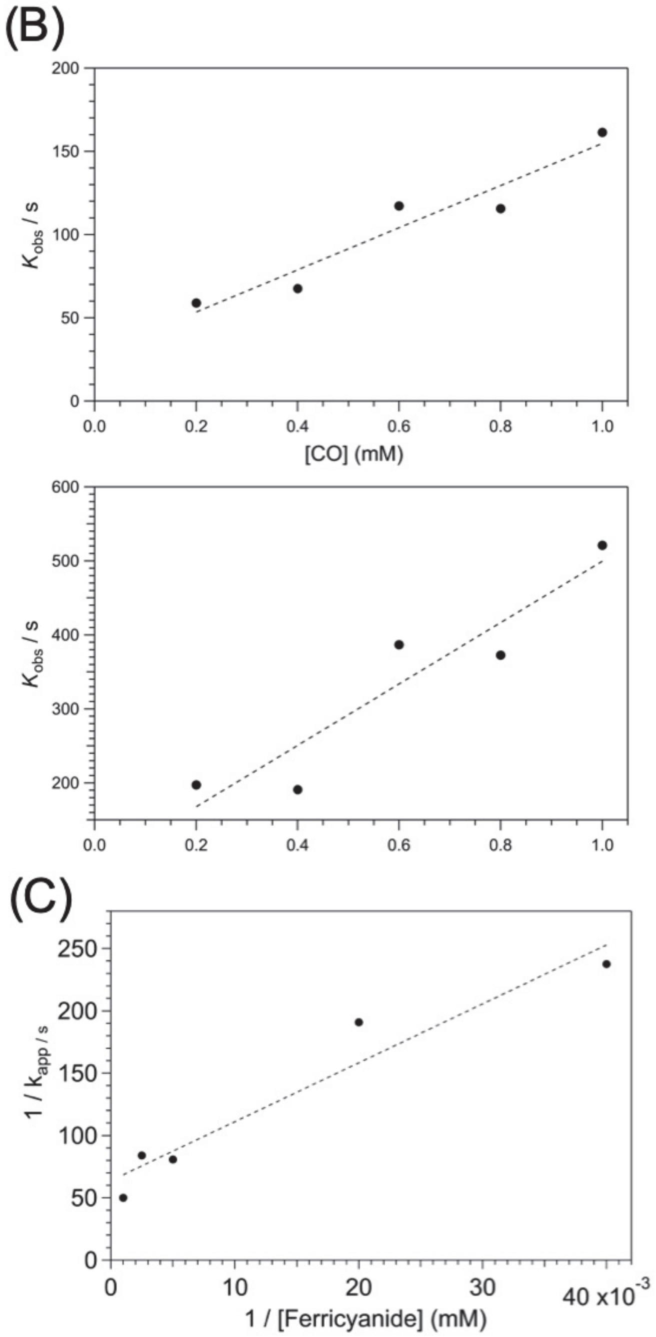

Supplementary Figure 12: CO kinetics of PefR. (A) Time course for CO dissociation and rebinding at different CO concentrations. *Left panel;* CO was photodissociated using a pump laser. The time course for differences in the absorbance at 420 nm before and after the photodissociation of CO from PefR (5 μM) was recorded for each CO concentration. *Right panel;* each curve was well fitted by a double exponential and the rate constant was calculated on the basis of the fitting equation. *Top of right panel;* Residual of the fitting. (B) CO dependence of two k_{obs} data sets for CO association kinetics from flash photolysis. The two determined k_{obs} data sets, K1 (top panel) and K2 (bottom panel), were plotted against CO concentrations. Each k_{on} value was determined from the slope of the fitted line. (C) Double reciprocal plot of the apparent rate constant for the CO dissociation reaction of holo-PefR. CO-bound holo PefR (5 μM) was maintained under anaerobic conditions. Various concentrations of potassium ferricyanide were added to oxidize heme iron and rapidly release CO. The attenuation curve of the time course for absorbance at 420 nm was measured and showed first-order decay. The apparent rate constant for CO dissociation (k_{app}) was calculated from the decay curve. The reciprocal of k_{app} for CO dissociation was plotted against the reciprocal of the concentration of potassium ferricyanide, and the *Y*-intercept corresponds to $1/k_{\mathrm{off}}$.

Supplementary Table 1: Rate constants for CO binding to various heme binding proteins

Protein	k_{on} ($\mu M^{-1}s^{-1}$)	k_{off} (s^{-1})	K_d (μM)	References
PefR	0.13/0.41	0.014	0.11/0.033	This work
Sperm whale myoglobin	0.51	0.019	0.037	3
Human hemoglobin α-chain	2.9	0.0046	0.0016 [a]	4
Human hemoglobin β-chain	7.1	0.0072	0.0010 [a]	4
Human cytoglobin	5.6	0.0030	n.d.[c]	5
Human neuroglobin	50/65	0.014	0.00021	6, 7
Rat heme oxygenase-1	1.3/0.31	0.0090	0.0067 [a] /0.029 [a]	8
CooA + DNA	32[b]	0.021	0.00066	9
CooA – DNA	24[b]	0.023	0.00096	9
RcoM-2	0.016	0.000064	0.0040 [a]	10
SUR2A	0.17	0.05	0.6	11

[a] Values are not given in the reference and were determined by dividing k_{on} by k_{off}.

[b]Values for the fastest and most abundant phase of the three phases were taken.

[c]No data.

References in Supplementary Information

1. Thompson, J. D., Higgins, D. G., Gibson, T. J. CLUSTAL W: improving the sensitivity of progressive multiple sequence alignment through sequence weighting, position-specific gap penalties and weight matrix choice. *Nucleic Acids Res.* **22**, 4673-4680 (1994).

2. Gouet, P., Courcelle, E., Stuart, D. I., Métoz, F. ESPript: analysis of multiple sequence alignments in PostScript. *Bioinformatics* **15**, 305-308 (1999).

3. Springer, B. A., Sligar, S. G., Olson, J. S., Phillips, G. N. Mechanisms of ligand recognition in myoglobin. *Chem. Rev.* **94**, 699–714 (1994).

4. Mathews, A. J., Rohlfs, R. J., Olson, J. S., Tame, J., Renaud, J. -P., Nagai, K. The effects of E7 and E11 mutations on the kinetics of ligand binding to R state human hemoglobin. *J. Biol. Chem.* **264**, 16573-16583 (1989).

5. Trent, J. T., Hargrove, M. S. A ubiquitously expressed human hexacoordinate hemoglobin. *J. Biol. Chem.* **277**, 19538–19545 (2002).

6. Uzan, J., Dewilde, S., Burmester, T., Hankeln, T., Moens, L., Hamdane, D., Marden, M. C., Kiger, L. Neuroglobin and other hexacoordinated hemoglobins show a weak temperature dependence of oxygen binding. *Biophys. J.* **87**, 1196–1204 (2004).

7. Dewilde, S., Kiger, L., Burmester, T., Hankeln, T., Baudin-Creuza, V., Aerts, T., Marden, M. C., Caubergs, R., Moens, L. Biochemical characterization and ligand binding properties of neuroglobin, a novel member of the globin family. *J. Biol. Chem.* **276**, 38949–38955 (2001).

8. Migita, C. T., Matera, K. M., Ikeda-Saito, M., Olson, J. S., Fujii, H., Yoshimura, T., Zhou, H., Yoshida, T. The oxygen and carbon monoxide reactions of heme oxygenase. *J. Biol. Chem.* **273**, 945–949 (1998).

9. Uchida, T., Ishikawa, H., Takahashi, S., Ishimori, K., Morishima, I., Ohkubo, K., Nakashima, H., Aono, S. Heme environmental structure of CooA is modulated by the target DNA binding. Evidence from resonance Raman spectroscopy and CO rebinding kinetics. *J. Biol. Chem.* **273**, 19988–19992 (1998).

10. Salman, M., Villamil Franco, C., Ramodiharilafy, R., Liebl, U., Vos, M. H. Interaction of the full-length heme-based CO sensor protein RcoM-2 with Ligands. *Biochemistry* **58**, 4028–4034 (2019).

11. Kapetanaki, S. M., Burton, M. J., Basran, J., Uragami, C., Moody, P. C. E., Schmid, R., Davies, N. W., Dorlet, P., Vos, M. H., Storey, N. M., Raven, E. A mechanism for CO regulation of ion channels. *Nat. Commun.* **9**, 907 (2018).
