# Supplementary material for: Heme controls the structural rearrangement of its sensor protein mediating the hemolytic bacterial survival
Source: Commun Biol. 2021 Apr 13;4:467. doi: 10.1038/s42003-021-01987-5 (PMC8044140; doi:10.1038/s42003-021-01987-5)
Supplement: Supplementary file 8 — Description of Additional Supplementary Files [file 42003_2021_1987_MOESM8_ESM.pdf]

Description of Additional Supplementary Files

File name: Supplementary Movie 1

Description: Morph movie (side view) of the conformational change between holo PefR and apo PefR-DNA complex.

File name: Supplementary Movie 2

Description: Morph movie (top view) of the conformational change between holo PefR and apo PefR-DNA complex.

File name: Supplementary Data 1

Description: Supplementary Data 1: All source data underlying Figures. Each data was separated on sheets of the Excel file.

File name: Supplementary Data2

Description: DNA (A) and protein (B) sequences of PefR, oligo primer sequences (C) and DNA sequencing data (D) for the site-directed mutagenesis. Sequences of (A)-(C) were summarized in the first sheet, (D) was separated from the second sheet by each DNA sequencing.
